# Association of daily selenium intake and glaucoma: A cross-sectional study

**DOI:** 10.1097/MD.0000000000039573

**Published:** 2024-09-06

**Authors:** Rumin Zhao, Tianwei Liu, Bojun Zhao

**Affiliations:** a Shandong University of Traditional Chinese Medicine, Jinan, Shandong Province, China; b Shandong Lunan Eye Hospital, Linyi, Shandong Province, China; c Cheeloo College of Medicine, Shandong University, Jinan, Shandong Province, China; d Shandong Provincial Hospital, Jinan, Shandong Province, China.

**Keywords:** dietary selenium, glaucoma, National Health and Nutrition Examination Survey (NHANES)

## Abstract

The association between dietary selenium intake and glaucoma remains unclear. Using data from the 2005 to 2008 National Health and Nutrition Examination Survey (NHANES), this study aimed to investigate the relationship between glaucoma and dietary selenium intake. The study included subjects aged 40 years or older who participated in the NHANES dietary intake interview and vision health questionnaire. Diagnosis of glaucoma based on self-reporting or fundus imaging. To investigate the relationship between daily selenium consumption and glaucoma, logistic regression analyses were employed. The potential linear relationship was found using smooth curve fitting. Subgroup analyses were also used. We found higher dietary selenium intake was associated with an increased risk of glaucoma (odds ratio, 1.39; 95% confidence intervals,1.07–1.81) on multivariable analysis. A linear association was found between dietary selenium intake and the occurrence of glaucoma in this population (*P*_non-linearity_ = 0.951). Subgroup analyses showed a stable correlation between dietary selenium intake and the occurrence of glaucoma (all *P* for interaction > .05).This is the first study to look at the connection between dietary selenium intake and glaucoma based on the data from the 2005 to 2008 NHANES. Our findings suggested that dietary selenium intake maybe positively correlated with the risk of glaucoma in adults older than 40 years old. To find out the potential relationship between dietary selenium intake and glaucoma, More longitudinal studies are required.

## 1. Introduction

Glaucoma is a chronic, progressive eye condition characterized by anatomical abnormalities of the optic nerve head and loss of visual field. It is the leading cause of irreversible blindness worldwide, and its prevalence is estimated to affect 76 million people worldwide.^[1,2]^ Intraocular pressure (IOP) is the only known modifiable risk factor for glaucoma, hence lowering IOP is the primary objective of all available medicinal and surgical treatments. However, glaucoma can occur even in the presence of low or normal IOP and can continue to deteriation. The etiology and pathogenesis of this disease remain unclear.^[3,4]^ Patients with glaucoma will benefit from the identification of additional modifiable risk factors.

In previous studies, researchers have shown the important role of nutrients and functional foods in preventing various chronic diseases.^[5]^ Selenium, which is present in many foods, is a trace element that is necessary for human health (lowering the chance of developing prostate, lung, and colon cancer) in very small amounts.^[6,7]^ After ingestion, selenium is integrated into several selenoproteins, such as glutathione peroxidases, thioredoxin reductases, iodothyronine deiodinases, and selenoprotein P, which mediate the biological effects of selenium.^[8]^ Although selenium is vital for human health, it is widely recognized that excessive selenium consumption has deleterious consequences on both people and animals.^[9–15]^ Numerous selenium-related effects, such as increased apoptosis and inhibition of vascular endothelial growth factor, neo-angiogenesis, and matrix metalloproteinases, limit the spread of cancer because they stop tumor growth and metastasis, but they are toxic to healthy tissue and impair normal cellular function.^[16–21]^

There are consistent signs of a link between elevated selenium levels and the risk of glaucoma in a selenium supplementation experiment conducted at the University of Arizona.^[22]^ However, according to a previous study, there was no difference in the amounts of selenium in the aqueous humor between patients with open-angle glaucoma and controls.^[23]^ Selenium’s on pseudoexfoliation glaucoma was also studied, and the results revealed no appreciable change in plasma selenium levels between cases and controls.^[24]^ These findings indicate that the effect of selenium on glaucoma remains inconclusive and remains a topic of investigation.

This study aimed to determine the dose–response relationship between dietary selenium intake and the occurrence of glaucoma using the National Health and Nutrition Examination Survey (NHANES), a large population-based study conducted in the United States (US).

## 2. Methods

### 2.1. Sample and population

Data from the 2005 to 2008 NHANES, a cross-sectional survey of civilian, noninstitutionalized Americans, was used to study the relationship between selenium intake and glaucoma.

All NHANES participants agreed to complete the measurements. Every NHANES cycle was a separate, cross-sectional survey. The National Center for Health Statistics Research Ethics Review Board approved the NHANES survey protocol. All participants provided written informed consent.^[25]^ The years 2005 to 2008 were chosen because surveys conducted during that period included questions and information about the presence or absence of glaucoma.

The main result was the presence or absence of glaucoma as defined by the disc or self-reported. Seven thousand eighty-one subjects who completed a dietary interview and were 40 years of age or older were included. Of the eligible subjects, 639 and 31 were eliminated because their dietary recall status was unreliable or they did not meet the minimum requirements, and because they did not answer the glaucoma interview question. Subjects who completed the visual questionnaire for the analysis of self-reported glaucoma outcomes and the retinal imaging examination for the investigation of image-based glaucoma outcomes were eligible after exclusion.

**Figure 1.**
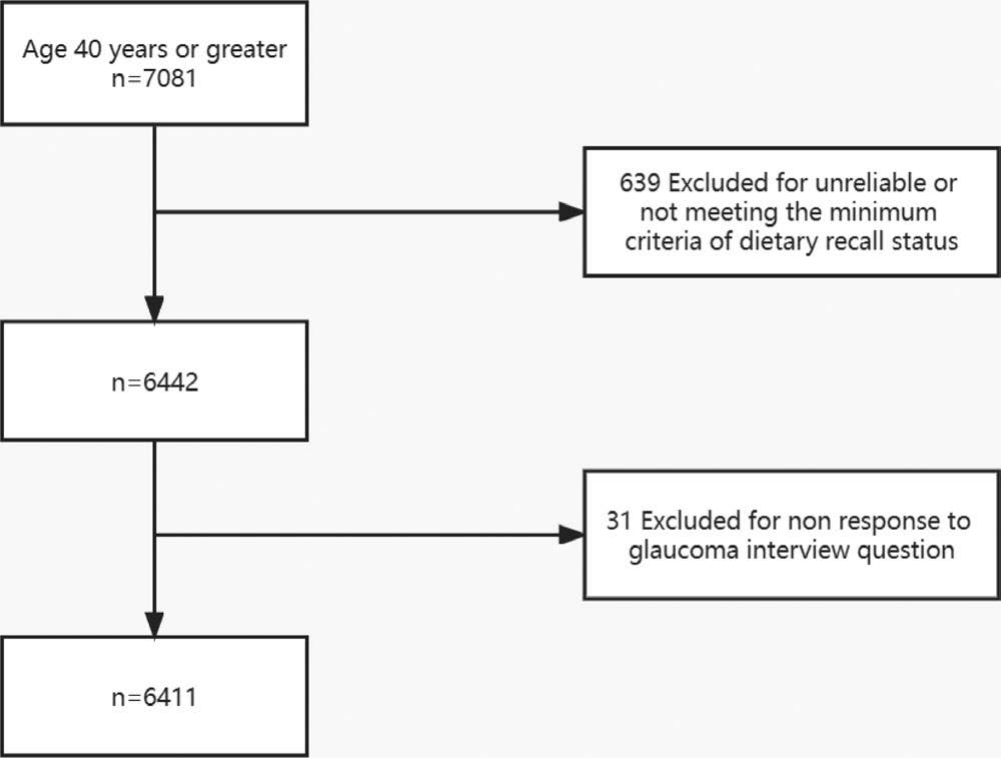
Flow diagram illustrating selection of study participants.

A total of 6411 subjects remained for the analysis of this outcome variable. Figure 1 shows a flow diagram that depicts a portion of the study participants.

### 2.2. Measures

Daily selenium intake, which was available in the “Dietary Interview-Total Nutrient Intakes dataset,” served as a predictor. Nutritional information was gathered from the What We Eat in America(WWEIA) dietary interview component. This survey was carried out in cooperation with the US Department of Agriculture (USDA) and the US Department of Health and Human Services. In accordance with this partnership, the USDA’s Food Surveys Research Group was in charge of the dietary data collection methodology, and the databases were used to code and process the data, while Department of Health and Human Services NCHS was in charge of sample design and data collection. All eligible NHANES examinees were given 2 24-hour dietary recall interviews to ask about the types and amounts of food they ate the day before the interview. The first dietary recall interview took place in person at the Mobile Examination Center, and the second one was conducted over the phone 3 to 10 days later.^[26]^

A summary of each person’s total nutritional consumption was given by the total nutrient intake dataset. The number of days of total intake, the days of the week of intake, and the daily energy totals were all included in each total intake record. Using the USDA’s Food and Nutrient Database for Dietary Studies (FNDDS), the nutritional contents of every food were determined. Every 2-year WWEIA/NHANES release cycle, the FNDDS values were revised. FNDDS 4.1 matched the 2007 to 2008 WWEIA. FNDDS 3.0 matched the 2005 to 2006 WWEIA.^[25,26]^

### 2.3. Outcome variables

In this study, fundus imaging (disc-defined glaucoma) and participant self-reports (self-reported glaucoma) were used to define glaucoma. The following question from the vision questionnaire, which was given to those over 40, was used to gather data for the assessment of self-reported glaucoma: “Have you ever been told by an Ophthalmologist that you have glaucoma, sometimes referred to as high pressure in your eyes?” The “Don’t Know” and “No Answer” respondents were not included in the analysis. Disc defined glaucoma was based on grading of Optic Disc Photographs. The optic nerve was imaged using a non-mydriatic fundus camera. (CR6-45NM; Canon USA, Melville, NY). Technicians were trained to determine when an image should be repeated, such as an image being too dark or the person blinked. After taking the required images, repeat images were captured for those that were obviously ungradable.

The University of Wisconsin Fundus Photograph Reading Center performed the initial grading of cup-to-disc ratio (CDR).^[26]^ Retinal images of all examined participants aged 40 years and older who had a vertical CDR 0.6 or greater were regraded in 2012 with attention to features relevant to glaucoma. Three glaucomatous experts (DSF, MVB, and PR) from the Wilmer Eye Institute registered everyone who had a CDR ≥ 0.6 in at least one eye according to the reading center. At the Wilmer Eye Institute, the images were graded to determine image quality, vertical CDR, notching of the neuro-retinal rim, excavation of the optic cup, optic disc hemorrhage, tilting of the disc, and disc size using a tablet-based review system (TruthMarker; IDx, LLC, Iowa City, IA). The likelihood of glaucoma (No, Possible, Probable, Definite, Unable) was rated on each image by 3 glaucomatous specialists.^[27]^ Glaucoma was deemed to be present in a participant if the consensus expert grade in either eye was “Probable” or “Definite.”

### 2.4. Covariates

Multiple confounders were included that could potentially impact the outcome, including:1. age (≥40 years), sex (male or female), race (non-Hispanic white, non-Hispanic black, Mexican and Hispanic, other races), education (less than high school, high school, more than high school), marital status (with partner, without partner), which were obtained through computer-assisted face-to-face interviews and can evaluate the social status and living status of participants; 2. diabetes (yes, no/unknown), energy, total folate, magnesium, sodium intake and BMI, which are all risk factors for glaucoma, therefore, they were also included as covariates in this study. Furthermore, each participant’s definition of diabetes was derived from the self-diagnosis of the clinicians. Please see www.cdc.gov/nchs/nhanes/ for additional information on dietary selenium consumption and glaucoma, as well as the procedure for acquiring other covariates.

### 2.5. Statistical analysis

Means and standard deviations for continuous variables and proportions for categorical variables were used to compute descriptive statistics. Using chi-square tests for categorical factors and t-tests for continuous variables, respectively, the distribution of potential confounding variables across participants with and without glaucoma was compared. Using the Taylor linearization approach, the population estimates’ standard errors were computed.

A multivariate logistic regression model was created to calculate odds ratio with 95% confidence interval for the relationship between dietary selenium intake and glaucoma. We either entered the covariates one by one into the basic model or removed them all from the complete model, then we compared the regression coefficients to control confounding factors. An unadjusted model is a crude model. Age, sex, and race were modified in Model I. Model II was adjusted for socio-demographic characteristics and some factors where *P* values were <.05, including age, sex, race, education level, marital status, diabetes, energy, total folate, magnesium intake, sodium intake and BMI.

A smooth curve fitting was employed to explore the dose-response relationship between dietary selenium and prevalence of glaucoma. Analysis of variance was used to assess for nonlinearity. Then a *P* for nonlinearity was calculated to detect linear trends (*P*_non-linearity_ > .05).

Additionally, a stratified logistic regression model was used to analyze the subgroups to further assess the stability of the results. To control for the effect of confounding factors, we choose to adjust for age, gender, race, education level, marital status, energy, total folate, magnesium, sodium, BMI, and diabetes. We conducted subgroup analyses of the covariates’ age, sex and diabetes status, which were key influencing factors of glaucoma. For the continuous variable age, we transformed it into categorical variable based on clinical cut points and then performed an interaction test. The likelihood ratio test was followed by tests for effect modification for those subgroup indicators. All *P* for interaction > .05 indicate that the correlation between dietary selenium intake and glaucoma was robust in different subgroups.

The statistical significance threshold was established at *P* < .05, and all tests were two-sided. This part of the analysis was performed using the statistical software packages R (http://www.R-project.org, The R Foundation, Ames, IA) and Free Statistics software versions1.7.1.

## 3. Results

### 3.1. Demographic characteristics of the study sample

A total of 6411 subjects aged 40 years or older who had accessible glaucoma interview responses and reliable selenium data were found in the NHANES data from 2005 to 2008. There were 431 self-reported defined glaucoma and 175 disc defined glaucoma. After deleting the overlap between these 2 criteria, 519 were glaucomatous. Among these defined glaucoma, 431 were self-reported, and 88 underwent fundus imaging.

Table 1 illustrates the demographic information, comorbidities, and health-related indicators of those with and without glaucoma. With the exception of selenium and total folate consumption levels, age, sex, race, education level, marital status, poverty, and other general health condition indicators (BMI, energy, protein, carbohydrate, sodium, and fiber intake) and with/without diabetes were not significantly associated with the prevalence of glaucoma.

**Table 1:** Comparison of demographics and characteristics of participants with or without glaucoma.

Characteristics	Total (n = 6411)	NO glaucoma	Glaucoma	*P* value
N	6411	5892	519	
Age (years)	60.3 ± 12.8	60.3 ± 12.8	59.7 ± 12.6	.311
*Gender*, N (%)				.946
Male	3190 (49.8)	2933 (49.8)	257 (49.5)	
Female	3221 (50.2)	2959 (50.2)	262 (50.5)	
*Race*/*ethnicity*, N (%)				.796
Non-Hispanic white	3381 (52.7)	3112 (52.8)	269 (51.8)	
Non-Hispanic black	1372 (21.4)	1252 (21.2)	120 (23.1)	
Mexican and Hispanic	1437 (22.4)	1324 (22.5)	113 (21.8)	
Other races	221 (3.4)	204 (3.5)	17 (3.3)	
*Education*, N (%)				.614
Less than high school	1983 (30.9)	1825 (31)	158 (30.4)	
High school graduation or equivalent	1568 (24.5)	1443 (24.5)	125 (24.1)	
More than high school	2855 (44.5)	2620 (44.5)	235 (45.3)	
NA	5 (0.1)	4 (0.1)	1 (0.2)	
*Marital status*, N (%)				.57
With partner	4006 (62.5)	3673 (62.3)	333 (64.2)	
Without partner	2402 (37.5)	2216 (37.6)	186 (35.8)	
NA	3 (0.0)	3 (0.1)	0 (0)	
A ratio of family income to poverty	2.7 ± 1.6	2.7 ± 1.6	2.7 ± 1.6	.836
BMI (kg/m^2^)	29.2 ± 6.5	29.2 ± 6.5	29.4 ± 6.4	.442
Energy (kcal)	1914.6 ± 791.4	1911.2 ± 791.7	1952.8 ± 787.7	.251
Protein (g/kg/d)	75.8 ± 33.6	75.6 ± 33.6	77.5 ± 33.2	.221
Carbohydrate (%energy)	233.8 ± 100.5	233.5 ± 100.8	236.7 ± 96.3	.493
Total folate (μg)	340.5 (247.0, 472.0)	339.5 (245.5, 470.0)	349.0 (261.2, 509.0)	.025
Fiber (g)	15.9 ± 8.3	15.9 ± 8.3	16.2 ± 8.5	.386
Magnesium (mg)	278.9 ± 122.2	278.0 ± 121.9	289.0 ± 125.6	.051
Selenium (μg)	101.6 ± 48.4	101.1 ± 47.9	106.5 ± 53.3	.016
Sodium (mg)	3071.5 ± 1438.7	3071.0 ± 1442.1	3077.7 ± 1400.0	.918
*Diabetes*, N (%)				.318
Yes	1415 (22.1)	1310 (22.2)	105 (20.2)	
No	4996 (77.9)	4582 (77.8)	414 (79.8)	

All eligible subjects had a mean daily selenium consumption of 101.6 ± 48.4 μg/day. The subjects with glaucoma (106.5 ± 53.3 μg/day) had significantly higher daily selenium consumption than those without glaucoma (101.1 ± 47.9μg/day) (*P* < .05).

### 3.2. Dietary selenium and glaucoma

The multivariate logistic regression models showed a positive correlation between glaucoma and dietary selenium intake. Table 2 shows the odds ratios and 95% confidence intervals for the 3 models. In model II, each per 100 μg increase in dietary selenium intake caused a 42% additional risk of glaucoma after adjustment for age, sex, race, marital status, education level, energy, total folate, magnesium, sodium, BMI, and diabetes.

**Table 2:** Association between dietary selenium intake and glaucoma in different models.

Dietary selenium	Crude Model		Model I		Model II	
	OR (95% CI)	*P*	OR (95% CI)	*P*	OR (95% CI)	*P*
Per 100 μg/d increment	1.24 (1.04,1.47)	.016	1.26 (1.05,1.53)	0.015	1.42 (1.08,1.86)	.012

Model I: adjusted for age, gender and race.

Model II: adjusted for Model I + education level, marital status, energy, total folate, magnesium, sodium, BMI, and diabetes.

CI = confidence interval, OR = odds ratio.

**Figure 2.**
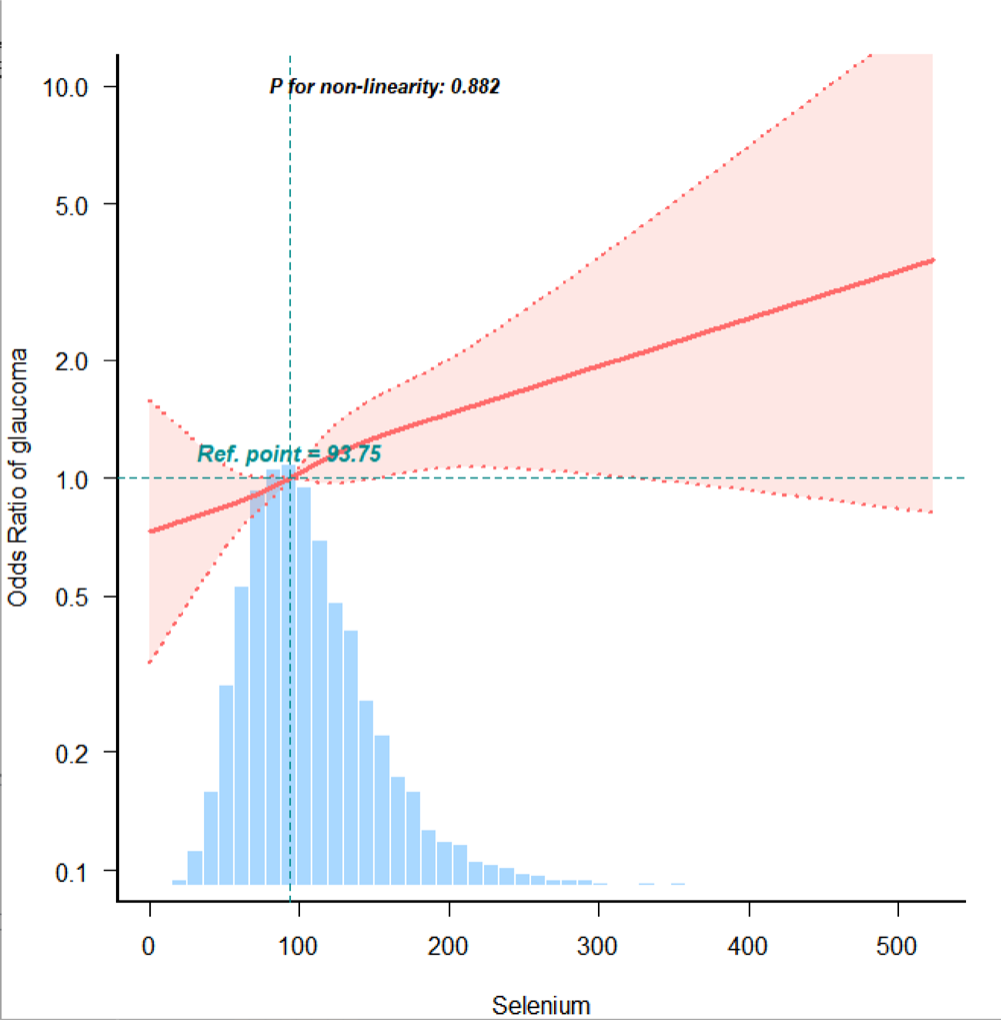
The dose–response relationship between dietary selenium intake and glaucoma. The solid line indicates the estimated risk of glaucoma, and the dotted lines represent a 95% confidence interval from the fit. Adjusted for age, gender, race, education level, marital status, energy, total folate, magnesium intake, sodium intake, BMI and diabetes.

Additionally, we utilized smooth curve fitting to demonstrate a positive linear relationship between dietary selenium intake and glaucoma within this population(*P* > .05, for nonlinearity) (Fig. 2), which was in line with the outcomes listed in Table 2.

### 3.3. Subgroup analyses

**Figure 3.**
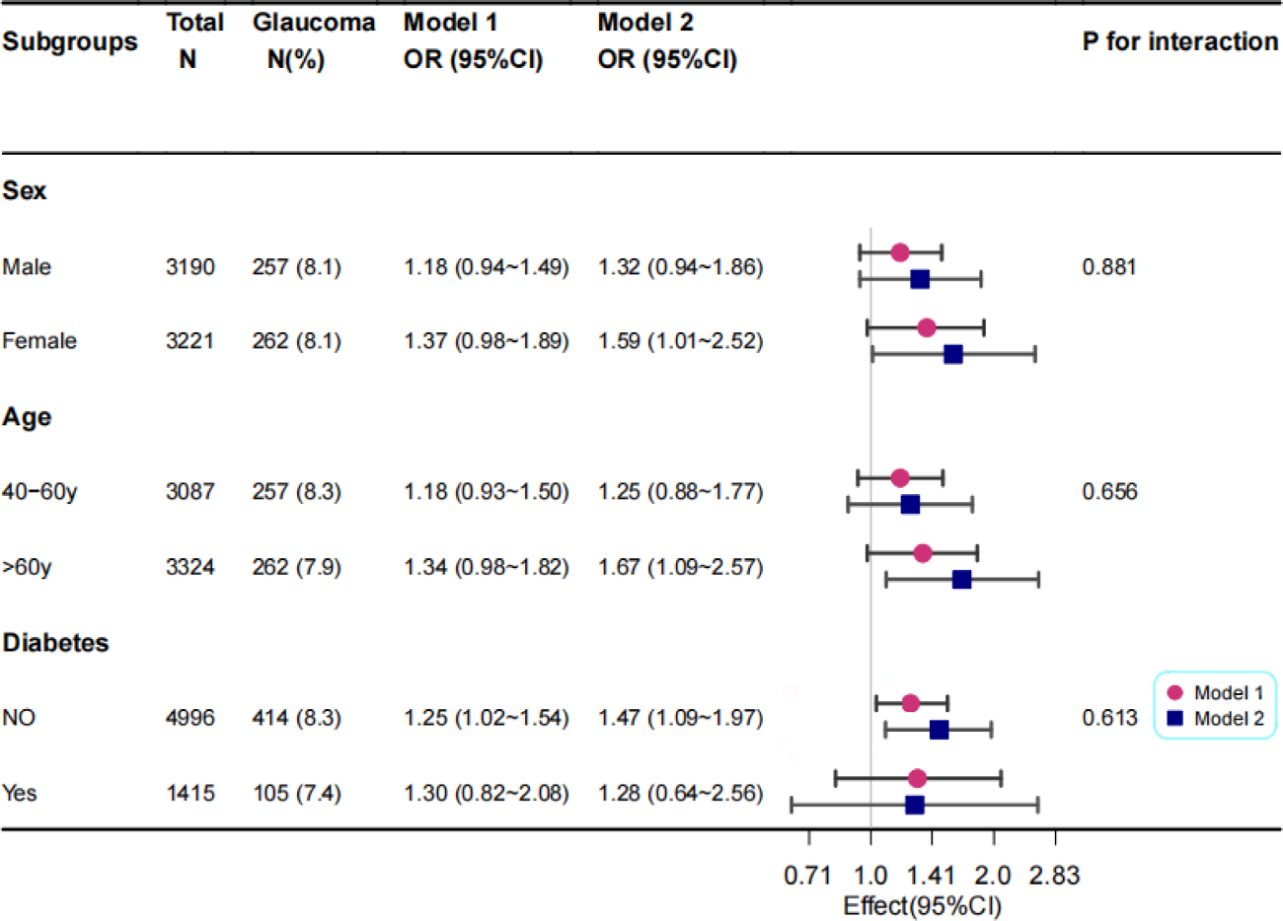
Association between selenium intake and glaucoma according to baseline characteristics. Model I: Adjusted for age, gender and race. Model II: Adjusted for Model I + education level, marital status, energy, total folate, magnesium intake, sodium intake, BMI and diabetes. CI = confidence interval, OR = odds ratio.

We further adopted a stratified analysis to assess whether the correlation between dietary selenium intake and glaucoma was robust in prespecified subgroups of key influencing factors (age, sex, and diabetes status) (Fig. 3). No significant interaction was observed in any of the subgroups (all *P* for interaction > .05), which indicated that the correlation between dietary selenium intake and glaucoma was robust in different subgroups.

## 4. Discussion

Glaucoma is a common condition, and the number of people with this condition has been increasing, which has a significant impact on health and medical care. It is still a global challenge because the pathogenesis is not very clear, and current treatments cannot prevent the progression of the disease. It is crucial to identify these affecting elements.

In this retrospective cross-sectional study, we first examined the association between dietary selenium intake and glaucoma based on data from the 2005 to 2008 NHANES. Multivariate logistic regression showed that higher dietary selenium intake was associated with an increased risk of glaucoma after adjusting for age, sex, race, education level, marital status, energy, total folate, magnesium, sodium, BMI and diabetes. In the meantime, after adjusting for confounding variables, the dose-response relationship between selenium intake and glaucoma was strongly positive in all participants. We found that the correlation between both was consistent and strong in different subgroup analyses.

Selenium is essential for human health and plays a significant role in gene expression and energy metabolism.^[28]^ The biological effects of selenium include anti-inflammatory, anti-oxidation, antiaging, and immune system control.^[29]^ For individuals, 55 to 79 g of selenium per day is the recommended dietary allowance. Intake of 30 to 40 μg of selenium per day is advised for children aged 4 to 13, while 60 μg or more per day is advised for women who are pregnant or nursing.^[30]^ A number of chronic metabolic disorders, including atherosclerosis, hyperglycemia, and hyperlipidemia, can be brought on by selenium deficiency.^[10]^ As a result, consistent selenium supplementation is required. However, when consuming too much selenium, symptoms of selenium poisoning can occur. Ataxia, diarrhea, vomiting, respiratory distress, and other symptoms are common in cases of acute selenium overdose. Persistent selenium toxicity is known to cause symptoms including sadness, exhaustion, garlicky breath odor, hair loss, and other issues.^[31]^ Thus, it is important to use dietary supplements correctly to avoid unneeded side effects.

Selenium maybe play a significant role in the pathogenesis of glaucoma with influence on human trabecular meshwork, according to research from the past. In, 2004M. Conley et al discovered that selenium-induced alterations in MMP-2/TIMP-1 secretion may disrupt the balance of extracellular matrix turnover in the traditional outflow channel and produce an increase in intraocular pressure that ultimately results in glaucoma.^[32]^ Elevated selenium levels were found to increase resistance to outflow in cultivated trabecular meshwork cells, according to an analysis of selenium’s effects.^[6]^ These findings showing a larger daily consumption of selenium may be linked to an increased incidence of glaucoma, which coincides with the results of our study.

Nevertheless, it is currently unclear how exactly dietary selenium levels relate to the development of glaucoma. According to numerous studies, oxidative stress could be the primary mechanism underlying the development of glaucoma.^[33,34]^ Selenium is a crucial part of the glutathione peroxidase complex, which controls oxidative stress.^[35,36]^ When oxidizing agents are active too much, it causes damage to biological components, which is called as oxidative stress. These oxidative effects have also been found to emerge as an important contributor to trabecular meshwork (TM) damage, particularly in the endothelial TM cells adjoining the Schlemm canal.^[37,38]^ Oxidative stress triggers apoptosis of TM cells, disrupts the extracellular matrix of the TM, and fosters TM cells fusion and excessive thickness. All of factors results in higher resistance to aqueous humor outflow, which raises the intraocular pressure.^[38–40]^

Our investigation has a number of advantages. First, the participants were representative of patients with glaucoma in the general US population. Second, the association between dietary selenium intake and glaucoma was estimated using dose–response analysis. Third, we performed a subgroup analysis to assess whether the correlation between dietary selenium intake and glaucoma was robust in the different subgroups. On the other hand, this study may have certain shortcomings. First, the results of our study do not support a cause-and-effect relationship between selenium intake and glaucoma owing to the observational nature of this study. Approximately 8.0% of the participants self-reported that they had glaucoma or ocular hypertension, which appears to be greater than the prevalence of glaucoma that has been previously reported. This may be attributed to the fact that more participants with ocular hypertension without glaucomatous changes were included in the NHANES study. Finally, there were unmeasurable confounding factors that could affect the accuracy of the conclusion, even though we tried to include as many potential confounding factors as possible.

## 5. Conclusion

This is the first study to look at the connection between dietary selenium intake and glaucoma based on the data from the 2005 to 2008 NHANES. Finding the link between dietary selenium consumption and glaucoma risk offers a great opportunity to lower glaucoma incidence and medical expenses and eventually enhance health results in elderly individuals. Our findings suggested that dietary selenium intake maybe positively correlated with the risk of glaucoma in adults older than 40 years old. To find out the potential relationship between dietary selenium intake and glaucoma, More longitudinal studies are required.

## Acknowledgments

We gratefully thank Jie Liu of the Department of Vascular and Endovascular Surgery, Chinese PLA General Hospital, for his contribution to the statistical support, study design consultations, and comments regarding the manuscript.

## Author contributions

**Conceptualization:** Rumin Zhao, Bojun Zhao.

**Formal analysis:** Tianwei Liu.

**Methodology:** Bojun Zhao.

**Writing – original draft:** Rumin Zhao.

**Writing – review & editing:** Bojun Zhao.
